# Supplementary material for: The interactome of intact mitochondria by cross-linking mass spectrometry provides evidence for coexisting respiratory supercomplexes
Source: Mol Cell Proteomics. 2017 Dec 8;17(2):216–32. doi: 10.1074/mcp.RA117.000470 (PMC5795388; doi:10.1074/mcp.RA117.000470)
Supplement: Supplemental Data [file supp_RA117.000470_133922_0_supp_23978_fzffwf.zip › spectra_annotation/mito_spectra_annotation/60-1-2-1-6-1.pdf]

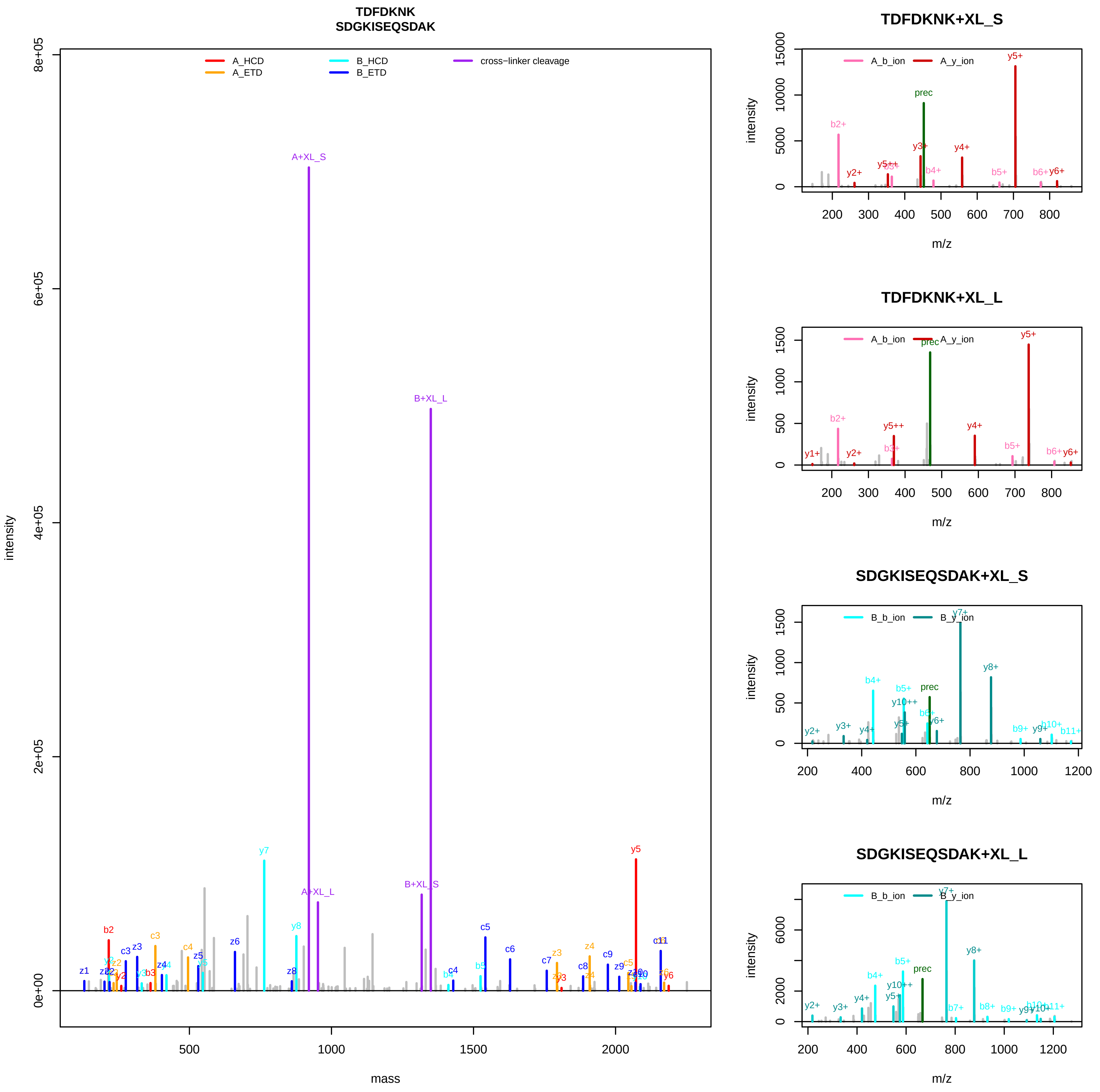
TDFDKNK
SDGKISEQSDAK
A_HCD
A_ETD
B_HCD
B_ETD
cross−linker cleavage
A+XL_S
B+XL_L
A+XL_L
B+XL_S
y7
y5
y8
b2
c3
z3
c4
z6
z5
c5
c6
c7
z3
z4
c9
c8
z9
z10
c10
c11
y6
b3
z4
z8
z1
y2
b4
c4
b5
intensity
mass
0e+00
2e+05
4e+05
6e+05
8e+05
500
1000
1500
2000
TDFDKNK+XL_S
A_b_ion
A_y_ion
prec
b2+
y2+
y3+
y4+
y5+
y5++
b3+
b4+
b5+
b6+
y6+
intensity
m/z
0
5000
10000
15000
200
300
400
500
600
700
800
TDFDKNK+XL_L
A_b_ion
A_y_ion
prec
b2+
y1+
y2+
y5++
b3+
y4+
b5+
y5+
b6+
y6+
intensity
m/z
0
500
1000
1500
200
300
400
500
600
700
800
SDGKISEQSDAK+XL_S
B_b_ion
B_y_ion
y7+
y8+
b4+
b5+
prec
y10++
b6+
y6+
y5+
y2+
y3+
y4+
b9+
y9+
b10+
b11+
intensity
m/z
0
500
1000
1500
200
400
600
800
1000
1200
SDGKISEQSDAK+XL_L
B_b_ion
B_y_ion
y7+
y8+
b5+
prec
b4+
y10++
y4+
y5+
y2+
y3+
b7+
b8+
b9+
y9+
b10+
b11+
intensity
m/z
0
2000
6000
200
400
600
800
1000
1200
